# Regulation of Viral Infection in Diabetes

**DOI:** 10.3390/biology10060529

**Published:** 2021-06-13

**Authors:** Seiho Nagafuchi

**Affiliations:** Department of Internal Medicine, Division of Metabolism and Endocrinology, Faculty of Medicine, Saga University, Saga 849-8501, Japan; su2733@cc.saga-u.ac.jp

Though there is no ‘Diabetes Virus’, multiple agents such as mumps virus, rubella virus, influenza virus, type A hepatitis virus, enterovirus, rotavirus, cytomegalovirus, varicella-zoster virus, human herpesvirus 6, Epstein-Barr virus, and also SARS-CoV-2 have been reported to be associated to diabetes [1,2]. Diabetes is a rather rare event among the large numbers of persons that become naturally infected by the above viruses. It is well ascertained, however, that the development of diabetes does depend on the diabetogenic potential of virus itself and on multiple risk factors of the host [3,4,5,6,7,8,9,10]. For the viral side, diabetogenicity, virus dose, route of inoculation or of natural infection, the consequent acute, persistent, or latent mode of infection are relevant factors [2,3,4,5,6]. Importantly, host resistance factors of the innate and adaptive immune responses have been recognized to play a central role [2,5,6,7,8,9,10]. In addition, the expression levels of viral receptors and of host molecules implicated in virus replication are connected with diabetes susceptibility [2]. In autoimmune type 1 diabetes, factors favoring virus-triggered autoimmunity to pancreatic β-cells undoubtedly play a pathogenic role [10]. In fulminant diabetes, the virus-host interaction appears decisive and apparently does not require an autoimmune response [11].

In susceptible strains of mice, an antecedent infection with the B variant of encephalomyocarditis virus (EMC) does prevent diabetes caused by the highly diabetogenic D variant of EMC [3,12]. In humans, extensive epidemiologic studies together with the above evidence from EMC-infected animals has led to the development of a polyvalent coxsackievirus B vaccine for reducing the incidence of T1D in genetically predisposed children [13].

One crucial question for the treatment of type 1 diabetes in the early phases is whether the disease would ameliorate if persistent infection could be terminated. To this end, multiple anti-enterovirus drugs are expected in the near future [14,15,16]. Indeed, a Norwegian trial with antiviral drugs is currently ongoing in type 1 diabetes (DiViD intervention trial: EudraCT number 2015-003350-41). If promising, the results of the above trial might change the therapeutic strategies for type 1 diabetes.

The susceptibility to multiple viruses, the regulation of viral infection in type 1 diabetes, the host factors that make some individuals susceptible to virus-induced diabetes undoubtedly merit intensive research. Investigations of this type may also foster progress in other immune-mediated conditions. Thus, critically presenting the results of ongoing research on virus-related diabetes and endocrine diseases does represent the scope of a dedicated issue of *Biology*. I look forward to receiving your research and review articles.
